# Depletion of proteasome subunit PSMD1 induces cancer cell death via protein ubiquitination and DNA damage, irrespective of p53 status

**DOI:** 10.1038/s41598-024-58215-3

**Published:** 2024-04-05

**Authors:** Mi-Yeun Kim, Eun-Ran Park, Eung-Ho Cho, Sun-Hoo Park, Chul Ju Han, Sang-Bum Kim, Man Bock Gu, Hyun-Jin Shin, Kee-Ho Lee

**Affiliations:** 1 Division of Radiation Biomedical Research, Korea Institute of Radiological and Medical Sciences, Korea University, 75, Nowon-Ro, Nowon-Gu, Seoul, 01812 South Korea; 2 Department of Biotechnology, College of Life Sciences and Biotechnology, Korea University, Seoul, South Korea; 3 Department of Surgery, Division of Radiological and Clinical Research, Korea Institute of Radiological and Medical Sciences, Seoul, South Korea; 4 Department of Pathology, Division of Radiological and Clinical Research, Korea Institute of Radiological and Medical Sciences, Seoul, South Korea; 5 Department of Internal Medicine, Division of Radiological and Clinical Research, Korea Institute of Radiological and Medical Sciences, Seoul, South Korea

**Keywords:** Cancer, Cell biology

## Abstract

Hepatocellular carcinoma (HCC) is characterized by high incidence and fatality rates worldwide. In our exploration of prognostic factors in HCC, the 26s proteasome subunit, non-ATPase 1 (PSMD1) protein emerged as a significant contributor, demonstrating its potential as a therapeutic target in this aggressive cancer. PSMD1 is a subunit of the 19S regulatory particle in the 26S proteasome complex; the 19S particle controls the deubiquitination of ubiquitinated proteins, which are then degraded by the proteolytic activity of the complex. Proteasome-targeting in cancer therapy has received significant attention because of its practical application as an established anticancer agent. We investigated whether PSMD1 plays a critical role in cancer owing to its prognostic significance. PSMD1 depletion induced cell cycle arrest in G2/M phase, DNA damage and apoptosis of cancer cells, irrespective of the p53 status. PSMD1 depletion-mediated cell death was accompanied by an increase in overall protein ubiquitination. These phenotypes occurred exclusively in cancer cells, with no effects observed in normal cells. These findings indicate that PSMD1 depletion-mediated ubiquitination of cellular proteins induces cell cycle arrest and eventual death in cancer cells, emphasizing PSMD1 as a potential therapeutic target in HCC.

## Introduction

Liver cancer is the sixth most common cancer and has a high fatality rate, with a 5-year survival rate of only 20%, ranking fourth among cancer mortalities worldwide^1^. Over 80–90% of liver cancers are hepatocellular carcinoma (HCC) originating from hepatocytes^2^ Patients with HCC exhibit symptoms after the disease has advanced, leading to an unfavorable prognosis. HCC demonstrates a heterogeneous molecular pattern arising from various factors, and targeted treatments for advanced liver cancer are ineffective^3^. These factors also contribute to a poor prognosis. Given this background, identifying therapeutic targets for liver cancer remains crucial and requires extensive research.

The ubiquitin–proteasome system is a crucial proteolytic mechanism for physiological processes that ensures protein homeostasis by eliminating damaged and unnecessary ubiquitinated proteins. The 26S proteasome complex, which is a part of the ubiquitin–proteasome system, degrades ubiquitin-tagged proteins^4,5^. It comprises the 20S-core and 19S-regulatory particles^6^. The 19S regulatory particle plays a role in deubiquitination^7^ and transfers substrate proteins to the 20S core particle for degradation^6^. The 20S core particle is a cylindrical heptameric complex in which two α- and two β-rings are stacked^8^. The α-rings comprise seven different α components that lead the substrate proteins into the catalytic chamber formed by the β-rings^8^. The β-rings degrade the proteins via the action of multi-catalytic proteinases composed of seven different β components^4,9^. The 26S proteasome subunit is overexpressed in various human cancers^10–12^. The inhibition of proteasome function impairs the activity of tumor cells, and proteasome inhibition has consequently emerged as an anticancer therapeutic strategy^13,14^.

Among the 26S proteasome subunits, non-ATPase 1 (PSMD1) is an essential component that forms the base of the 19S regulatory particle^15–17^. PSMD1 is clinically associated with human cancers and cancer cell aggressiveness. Similarly to other 26S proteasome subunits, PSMD1 is overexpressed in human cancer cells^10,11,18,19^. Its overexpression has been reported to correlate with poor prognosis, with PCR analysis of patient tissues in gastric cancer^8^ and analysis utilizing a public resource of HCC^10,20^. Functionally, PSMD1 controls the proliferation and apoptosis of cancer cells^10^ by degrading NOXA^21^ and p53^18^. Although PSMD1 is a well-known component of the 19S regulatory particle, and its overexpression has been reported in some cancers, its intrinsic function or specific role in cancer has not been well studied. As several reports have demonstrated the effects of PSMD1 on cancer growth and prognosis, we assessed its role in human cancer cells. To ascertain the function of PSMD1 in cancer, we analyzed the expression and function of PSMD1 in cancer, focusing on liver cancer cells driven by distinctively increased expression and prognostic implications. We revealed that the function of PSMD1 as a proteasome subunit is critical for survival of cancer cells.

## Results

### PSMD1 is a prognostic factor and overexpressed in patients with HCC

**Figure 1:**
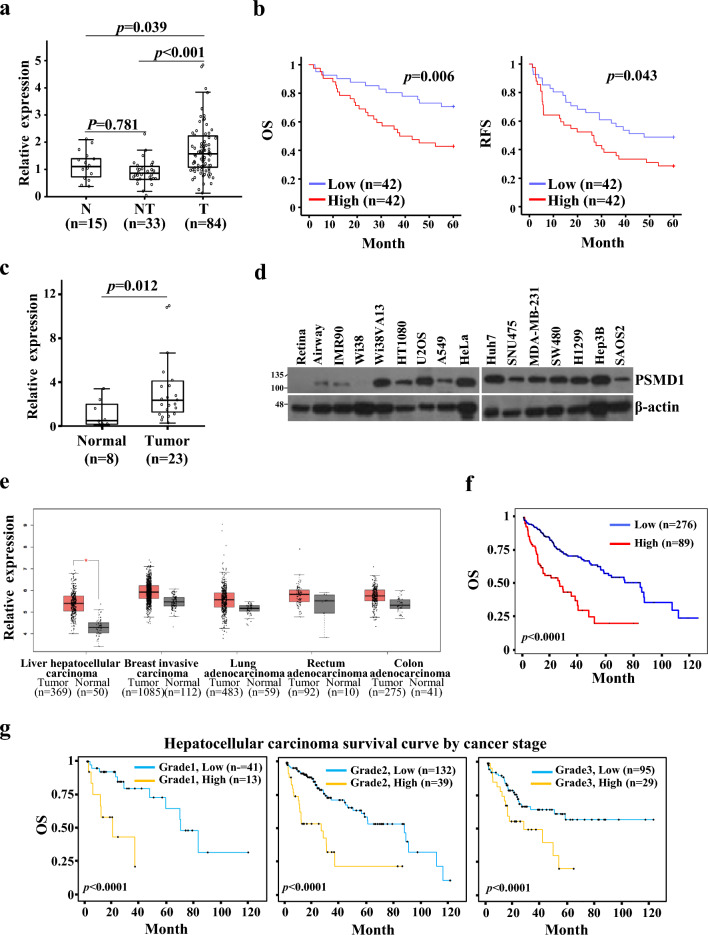
Analysis of PSMD1 expression in HCC tissues. The expression analysis in hepatocellular carcinoma tissue analysis was conducted using patient tissues obtained from the Korea Institute of Radiological and Medical Sciences (KIRAMS) (**a**,**b**), along with the purchase of cDNA (**c**) and utilizing information from The Cancer Genome Atlas (TCGA) (**e**–**g**). (**a**) Boxplot analysis depicting increased PSMD1 expression in HCC tissues (n = 84) compared with adjacent liver tissues (n = 33) and normal liver tissues (n = 15). (**b**) Kaplan–Meier survival analysis for PSMD1 expression in patients with HCC (left: overall survival, right: recurrence-free survival). (**c**) The relative expression of PSMD1 in human hepatocellular carcinoma and normal tissues was analyzed using a TissueScan cDNA array and real-time PCR. Normal tissues corresponded to non-tumor regions with normal limits, as assessed pathologically. (**d**) Expression of PSMD1 in various human normal and cancer cells was determined by western blot analysis using normal epithelial cells (retinal pigment epithelial cells, small airway epithelial cells), normal fibroblasts (Wi38 and IMR90), transformed fibroblasts (Wi38VA13), fibrosarcoma (HT1080), osteosarcoma (U2OS, SAOS-2), non-small cell lung cancer (A549, H1299), cervical cancer (HeLa), hepatocellular carcinoma (Huh7, SNU475, Hep3B), breast cancer (MDA-MB-231), and colorectal cancer (SW480). The results presented in two separate gels were obtained simultaneously under identical conditions. The increase of PSMD1 expression in cancer cell lines was consistently validated through three independent experiments. The results presented in two separate gels were obtained simultaneously under identical conditions. The increase of PSMD1 expression in cancer cell lines was consistently validated through three independent experiments. (**e**) The relative expression levels of PSMD1 in cancer (hepatocellular carcinoma, breast invasive carcinoma, and lung, rectum, and colon adenocarcinoma) and corresponding normal tissues were analyzed using the GEPIA platform. (**f**) Kaplan–Meier survival curves of patients with hepatocellular carcinoma were analyzed using the UALCAN platform based on the level of PSMD1 expression. (**g**) The survival curves were further analyzed for each tumor grade. Uncropped western blot images corresponding to Fig. 1 can be found in Supplementary Fig. S6.

To investigate the involvement of PSMD1 in the pathogenesis of HCC, we analyzed its expression in tumor tissue specimens obtained from patients who underwent surgical resection at the Korea Cancer Center Hospital, using real-time PCR on liver tissue samples (n = 84) (Table 1). We observed significantly higher *PSMD1* mRNA expression in HCC tissues than in adjacent non-tumor liver tissues (NT) (*p* < 0.001) or normal liver tissues (N) (*p* = 0.039) (Fig. 1a). Compared with normal liver tissues, there was a mean 1.84-fold increase in PSMD1 expression (median, 1.49-fold) in HCC tissues. To validate PSMD1 as a prognostic factor for HCC, Kaplan–Meier survival analysis was performed (Fig. 1b). The group with higher expression levels of PSMD1 (n = 42) exhibited decreased overall (*p* = 0.009) and recurrence-free survival rates (*p* = 0.043) compared to the group with lower expression levels (n = 42). To further assess PSMD1 expression in other datasets, we used commercially available hepatocellular carcinoma cDNA and measured mRNA levels. The relative *PSMD1* mRNA expression (*PSMD1*/β2-microglobulin) was significantly higher in hepatocellular carcinoma tissues (n = 23, 3.81 ± 4.36) compared with that of the corresponding normal tissues (n = 8, 1.10 ± 1.23; *p* = 0.012) (Fig. 1c). We analyzed the PSMD1 protein levels in normal and cancerous cell lines. Most of the tested human cancer cell lines from various origins (HT1080: fibrosarcoma, U2OS, SAOS-2: osteosarcoma, A549, H1299: lung cancer, HeLa: cervical cancer, Huh7, SNU475, Hep3B: hepatocellular carcinoma, MDA-MB-231: breast cancer, SW480: colorectal cancer) had higher levels of PSMD1 protein compared with that of normal human retinal pigment epithelial cells, small airway epithelial cells, and normal fibroblasts (Wi38 and IMR90) (Fig. 1d). Additionally, we analyzed the expression of PSMD1 in various human cancer tissues using The Cancer Genome Atlas (TCGA) database. PSMD1 expression was significantly increased in all analyzed cancer tissues, including HCC, breast carcinoma, and lung, rectum, and colon adenocarcinomas, compared with that of the corresponding normal tissues (Fig. 1e). The degree of increase in PSMD1 expression compared with that in normal tissues was particularly pronounced in HCC. We then analyzed the correlation between patient survival and PSMD1 expression in HCC from the TCGA database using the UALCAN platform. Kaplan–Meier survival curves showed that patients with PSMD1 overexpression had poorer overall survival rates (*p* < 0.0001) (Fig. 1f). The survival in the two expression groups was analyzed separately for tumor grades 1, 2, and 3. As shown in Fig. 1g, patients with high PSMD1 expression exhibited poor prognosis across all grades (*p* < 0.0001), and significantly lower survival rates were observed, even in grades 1 and 2. The survival curve was further analyzed using other cancers examined for expression (Fig. 1e; Supplementary Fig. S1. Although a poor prognostic factor was also confirmed in breast and lung cancers, the distinction was less pronounced than in liver cancer.

**Table 1:** Patient clinical pathologic data (n = 84).

Variables	Classification	Distribution
Gender	Male : Female	74 : 10
Age	Year, mean ± SD (range)	52.54 ± 10.65 (26–74)
Etiology	Hepatitis B : Hepatitis C	71 : 05
AST	IU/L, mean ± SD (range)	53.00 ± 36.44 (15–182)
ALT	IU/L, mean ± SD (range)	47.19 ± 25.31 (10–130)
AFP	< 100 ng/dL : 100 ng/dL≦	49 : 33
Prothrombin (%)	< 90 : 90 ≤	34: 49
Child–Pugh classification	A : B	68 : 07
Total bilirubin	< 1 mg/dL : 1 mg/dL ≤	54 : 29
Tumor size	cm, mean ± SD (range)	5.8 ± 3.29(1.2 ~ 15)
Tumor number	Single : Multiple	57 : 17
Tumor stage	I : II : III : IV	02 : 30 : 18 : 09
Cirrhosis	No : Yes	46 : 31
Macroscopic vascular invasion	No : Yes	73 : 5
Microscopic vascular invasion	No : Yes	34 : 37
Capsule invasion	No : Yes	43 : 39
Serosal invasion	No : Yes	66 : 12
Edmondoson grade^a^	I : II : III : IV	10 : 45 : 27 : 00

*AFP* α-fetoprotein, *AST* aspartate aminotransferase, *ALT* alanine transaminase.

^a^Edmonson-Steiner histological grade.

### PSMD1 expression is associated with α-fetoprotein (AFP) levels and tumor size

We investigated the association between PSMD1 mRNA levels and clinicopathological parameters to determine whether PSMD1 overexpression has clinical implications in HCC. In the analysis, clinical variables, including AFP level (< 100 vs. ≥ 100 ng/mL) (*p* = 0.001) and tumor size (< 7 vs. ≥ 7 cm) (*p* = 0.044) exhibited associations with PSMD1 expression (Table 2). Particularly, the association between PSMD1 and AFP levels was significant, irrespective of the AFP cutoff criteria (Table 3). In contrast, HCC-based variables such as gender (*p* = 0.369), age (*p* = 0.254), Child–Pugh classification (*p* = 0.515), tumor number (*p* = 0.5), microvascular invasion (*p* = 0.549), and Edmondson grade (*p* = 0.438) were not associated with PSMD1 overexpression (Table 2).

**Table 2:** The correlation between PSMD1 expression and clinicopathological parameters (n = 84).

Variables	TMEM165 Expression		*p*
	< 1.5 fold	≧1.5 fold	
Gender			
Male	38	36	0.369
Female	4	6	
Age (year)			
< 52	16	20	0.254
≥ 52	26	22	
AFP (ng/mL)			
< 100	32	18	**0.001**
≥ 100	9	24	
AST (U/L)			
< 40	20	18	0.413
≥ 40	22	24	
ALT (U/L)			
< 35	12	19	0.117
≥ 35	29	24	
Child–Pugh classification			
A	34	34	0.515
B	3	4	
Tumor size (cm)			
< 7	32	23	**0.044**
≥ 7	10	18	
Tumor stage			
I, II	18	14	0.113
III, IV	10	17	
Tumor number			
Single	29	28	0.5
Multiple	8	9	
Macroscopic vascular invasion			
No	38	36	0.488
Yes	2	3	
Microscopic vascular invasion			
No	17	17	0.549
Yes	19	18	
Edmondoson grade			
I, II	27	28	0.438
III, IV	15	13	
Cirrhosis			
No	24	22	0.355
Yes	14	17	

Significance of PSMD1 overexpression in association with clinicopathological parameters was calculated using Chi-Square Test.

Bold indicates p < 0.05. Statistically significant data in bold.

**Table 3:** The correlation between PSMD1 expression and AFP levels (n = 83).

AFP (ng/ml)	PSMD1 Expression		*p*
	< 1.5 fold	≧1.5 fold	
< 20	25	11	0.001
≥ 20	16	31	
< 50	28	17	0.010
≥ 50	13	25	
< 100	32	18	0.001
≥ 100	9	24	
< 200	33	20	0.002
≥ 200	8	22	
< 400	34	26	0.028
≥ 400	7	16	

### PSMD1 depletion increases cancer cell death and induces DNA damage

**Figure 2:**
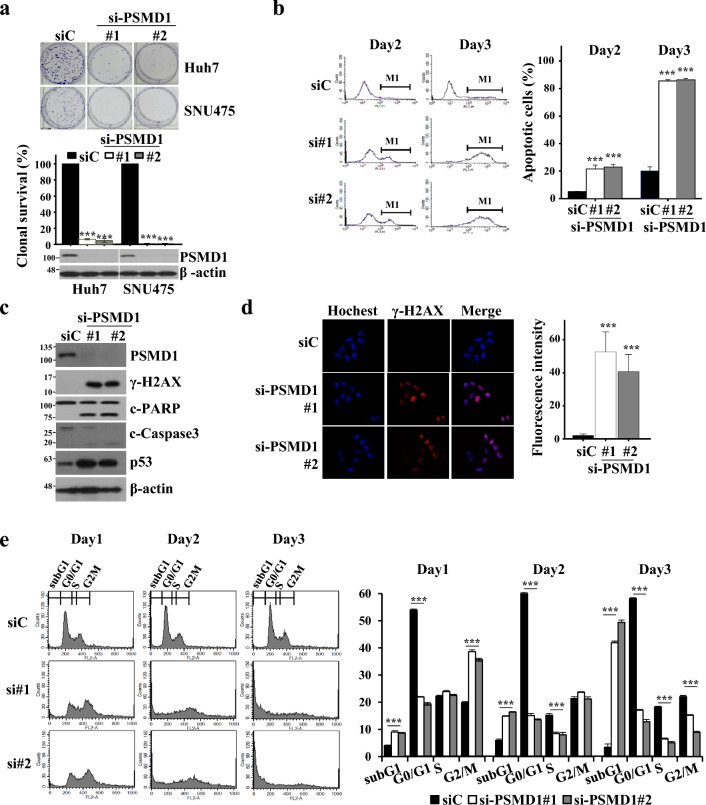
PSMD1 depletion increases DNA damage and apoptosis. (**a**) Clonal survival of Huh7 and SNU475 cells was analyzed by low-density seeding of cells transfected with control- (siC) and PSMD1-siRNA (#1 and #2). The survival rates were determined by comparing survived colonies transfected with control- and PSMD1-siRNA. (**b**) To analyze apoptosis, siRNA-transfected cells were stained with annexin V and subjected to FACS analysis. Apoptotic cells were determined by counting the annexin V-positive cells and presented as a bar graph. (**c**) Two days after the siRNA transfection in Huh7 cells, the protein levels of γ-H2AX, p53, cleaved PARP, and cleaved caspase 3 were determined using Western blotting. β-actin was used as an internal control. (**d**) Representative picture showing DNA damage 1 d after siRNA transfection. Nuclear DNA damage was monitored using an anti-γ-H2AX antibody and a red-fluorescent secondary antibody and visualized under a fluorescent microscope (× 40). Hoechst dye was used to counterstain the nuclei. The fluorescence intensity of individual cells was quantified using the ImageJ program. (**e**) To analyze the cell cycle, siRNA-transfected Huh7 cells were stained with propidium iodide and analyzed using FACS. The relative percentages of populations enriched at each cell cycle phase were determined from the histogram, and the means of independently prepared triplicates were depicted as a bar graph. *** indicates *p* < 0.001. Uncropped western blot images corresponding to Fig. 2 can be found in Supplementary Fig. S7.

To investigate the functional role of PSMD1, we selected HCC cell lines overexpressing PSMD1 and examined the survival of cancer cells under PSMD1 depletion. Both small interfering RNAs (siRNAs) targeting PSMD1 were confirmed to inhibit cancer cell survival by over 90% in Huh7 and and over 99% in SNU475, compared with control-siRNAs (Fig. 2a). To confirm whether the PSMD1 depletion-induced inhibition of clonal survival was due to apoptosis, we conducted annexin V staining of Huh7 cells with depleted PSMD1. There was an increase in the population strongly stained with annexin V in the fluorescence-activated cell sorting (FACS) histogram (M1 on the histogram, Fig. 2b).

Apoptosis was further validated using western blot analysis (Fig. 2c). Depletion of PSMD1 in Huh7 cells led to increased cleavage of poly ADP-ribose polymerase (PARP) and caspase 3, along with elevated expression levels of γ-H2AX and p53. The γ-H2AX expression was also verified by immunostaining. Upon transfection with PSMD1-siRNA, the staining intensity of the γ-H2AX protein increased in the nucleus (Fig. 2d). As γ-H2AX serves as a marker for apoptosis and is also a critical indicator of DNA damage, the increased γ-H2AX due to PSMD1 depletion indicates that severe DNA damage occurred. We further delineated the correlation between DNA damage and apoptosis following PSMD1 depletion by analyzing the cell cycle. PSMD1-depleted Huh7 cells exhibited a gradual increase in the subG1 population over time, indicating massive nuclear fragmentation, which is evidence of apoptotic death (Fig. 2e). Specifically, the G2/M population was dominant within 24 h. 72 h after siRNA transfection, most cells were in the subG1 phase, indicating that PSMD1-depleted cells were arrested in the G2/M phase and subsequently underwent apoptosis.

### PSMD1 depletion decreases the clonal survival of cancer cells, irrespective of p53 status

**Figure 3:**
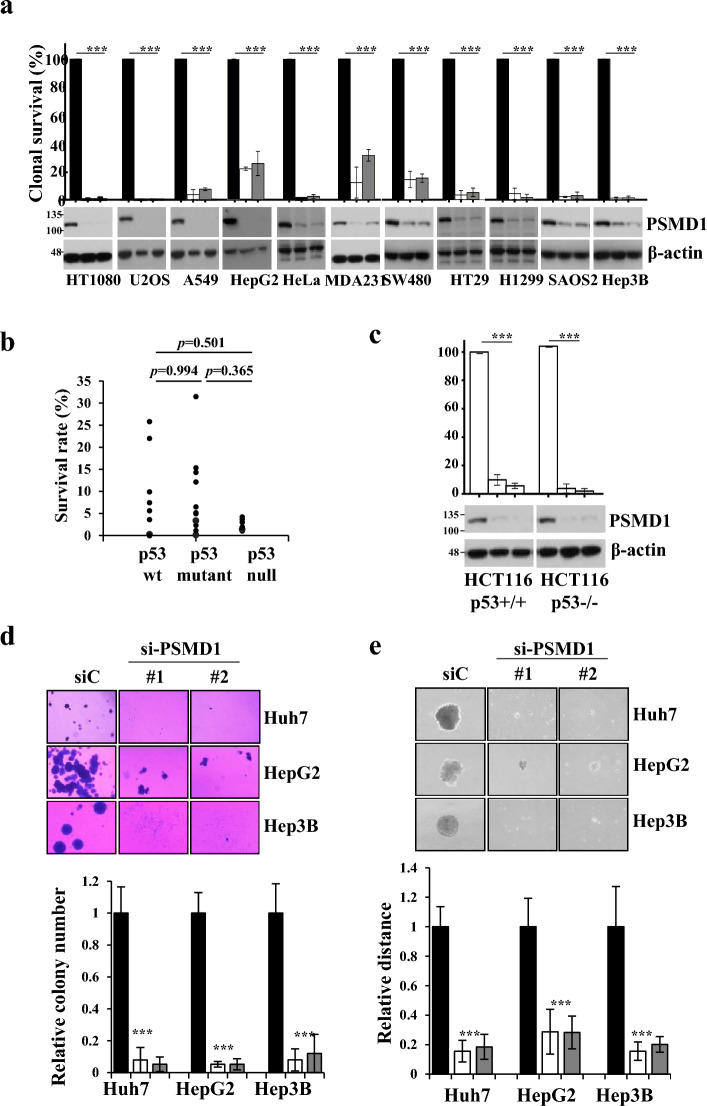
PSMD1 depletion weakens cancer cell progression activity irrespective of the p53 status. (**a**) The effect of PSMD1 depletion on cancer cell survival was determined using clonal survival assay using cancer cell lines with wild-type p53 (HT1080, U2OS, A549, and HepG2), inactive p53 (HeLa), mutant p53 (MDA-MB-231, SW480, and HT29) and null p53 (H1299, SAOS2, and Hep3B). (**b**) Clonal survival rates resulting from PSMD1 depletion compared with control (**a**) categorized by p53 status and presented using a spot graph. (**d**,**e**) The effects of PSMD1 depletion on anchorage-independent cell growth were determined using colony formation in soft agar (**d**) and spheroid formation on poly-HEMA-coated plates (**e**) in Huh7, HepG2, and Hep3B cells. Bar graphs depicting the relative colony number (**d**) and sphere diameter (**e**) of PSMD1-siRNA-transfected cells compared with control-siRNA-transfected cells were generated from triplicated data. Uncropped western blot images corresponding to Fig. 3 can be found in Supplementary Fig. S8.

PSMD1 depletion promotes cancer growth by inhibiting p53 degradation^18^. However, Huh7 and SNU475 HCC cells, which exhibit a significant reduction in cell survival upon PSMD1 depletion, coincidentally harbor p53 mutations (Huh7-Y220C, SNU475-G262D/N239D) that impair the function of p53^22,23^. For further validation, various cancer cell lines with varying p53 expression levels were used. For wild-type p53, we used HT1080, U2OS, A549, and HepG2. For mutant p53, we selected MDA-MB-231 (R280K), SW480 (R273H/P309S), and HT29 (R273H) cells that harbor hotspot mutations that inactivate p53^24^. Additionally, we included HeLa cervical carcinoma cells, which contain p53 inactivated by human papillomavirus protein. For null-type p53, H1299, SAOS2, and Hep3B cells were used. All tested cell lines showed severe decreases in clonal survival, regardless of the p53 status (Fig. 3a). Although there were differences in the degree of apoptosis among the cell lines, a trend according to p53 status was not observed (Fig. 3b). This was confirmed in HCT116 cells and genetically modified cells lacking p53 (Fig. 3c).

We further investigated the impact of PSMD1 on cancer progression using three HCC cell lines with different p53 statuses, examining the effect of PSMD1 depletion on anchorage-independent cell growth and assessing tumor aggressiveness^24^ through the analysis of soft agar colony formation and spheroid formation. All cell lines–Huh7, HepG2, and Hep3B–exhibited a significant decrease of cell growth on soft agar (Fig. 3d) and spheroid formation (Fig. 3e) upon PSMD1 depletion. These results indicate that PSMD1 depletion inhibits cancer cell survival and weakens cancer progression, irrespective of the p53 status.

### The severity of PSMD1 inhibition selectively operates more prominently in cancer cells than in normal cells

**Figure 4:**
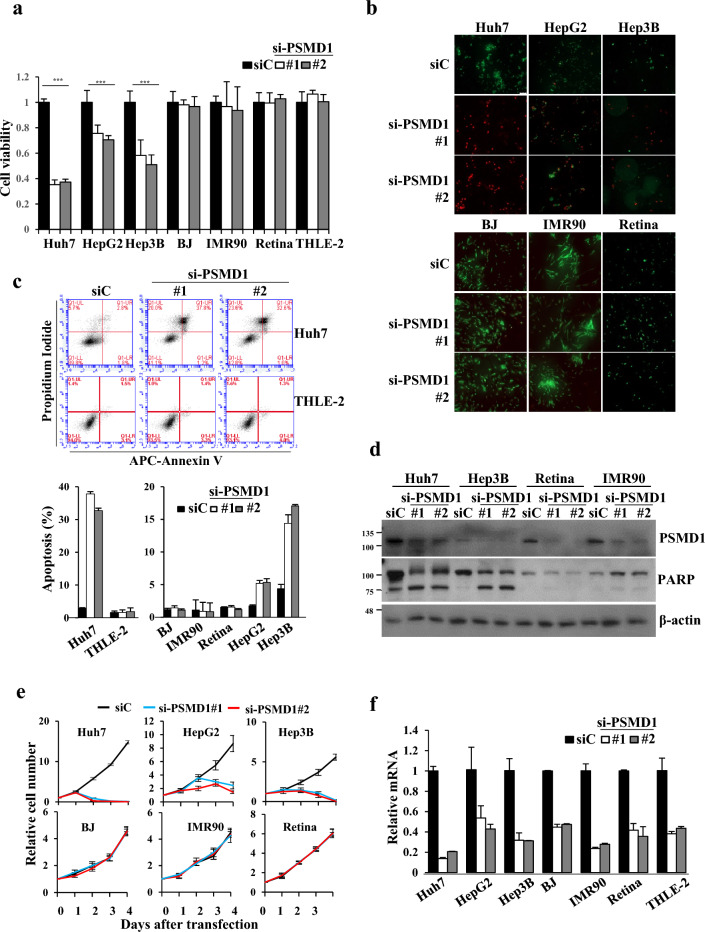
PSMD1 depletion-mediated survival inhibition occurs predominantly in cancer cells. (**a**–**e**) The effects of PSMD1 depletion by siRNA transfection on cell proliferation (CCK-8 assay) (**a**), cell death (live/dead assay) (**b**), apoptosis (annexin V/PI staining and western blotting) (**c**,**d**), cell growth (cell counting) (**e**) were compared among HCC cell lines and normal cells. **(a)** The mean optical density of 420 nm was compensated by subtracting the background optical density at 420 nm from the culture media sample represented as a bar graph. Relative values of optical density at 420 nm were depicted. (**b**) Merged images of stained cells. The green color indicates live cells, and the red indicates dead cells. (**c**) Apoptosis was analyzed and compared between Huh7 and THLE-2 and other indicated cells. Late apoptotic cells that stained both annexin V and PI were calculated as apoptotic cells from scatter plots with quadrants. (**d**) Western blotting was conducted on cell lysates harvested three days after transfection using the indicated antibodies. (**e**) 1 × 10^4^ cells seeded on six well plate were transfected at day 0 and cells were counted daily for 4 days. (**f**) The relative *PSMD1* mRNA levels in each cell line were determined using real-time PCR, normalized to β2M, and calculated using the 2^−ΔΔCt^ method. *** indicates *p* < 0.001. Uncropped western blot images corresponding to Fig. 4 can be found in Supplementary Fig. S9.

Because we identified PSMD1 as a poor prognostic factor overexpressed in HCC, we compared the effects of its expression inhibition in HCC cells with distinct p53 statuses (Huh7, HepG2, Hep3B) and in several normal cells containing immortalized normal epithelial liver cells (THLE-2) to determine whether the inhibition of cell survival through PSMD1 depletion is more sensitive in cancer. The effects of PSMD1 depletion on cells were analyzed using the Cell Counting Kit-8 (CCK-8) assay for cell viability (Fig. 4a), live/dead staining for cell death (Fig. 4b), annexin/propidium iodide (PI) staining (Fig. 4c, Supplementary Fig. 2b), and western blotting for apoptosis (Fig. 4d), cell counting for cell growth analysis (Fig. 4e) and γ-H2AX staining for detecting DNA damage (Supplementary Fig. S2a). All the tested normal cells (THLE-2, BJ, IMR90, and retinal) exhibited less impact on these phenotypes following PSMD1 depletion. In live/dead staining, after three days of transfection with PSMD1 siRNA (two days for Huh7 cells), the red signal indicating dead cells increased in cancer cell lines, whereas the green signal indicating live cells remained intact in normal cells (Fig. 4b). Apoptosis induced by PSMD1 depletion, as shown in Fig. 2, was further confirmed in additional HCC cells with different p53 statuses, as evidenced by increased annexin V/PI co-staining (Fig. 4c, supplementary Fig. 2b) and PARP protein cleavage (Fig. 4d), whereas normal cells did not exhibit apoptosis. DNA damage was also not observed in normal cells (Supplementary Fig. S2a). After allowing the cells to grow to confluence for five days following transfection, no cytotoxicity was observed (supplementary Fig. S2c). The absence of a discernible difference in cell growth rate between the control and PSMD1 siRNA, as determined by cell counting (Fig. 4e), indicated that the lack of impact on apoptosis or cell death in normal cells cannot be attributed to delayed cytotoxicity resulting from their slower response. To exclude the possibility that the lack of impact on normal cells of PSMD1 depletion was simply due to low transfection efficiency, knockdown efficiency was compared using real-time PCR among all tested cell lines. As shown in Fig. 4f, the effective reduction of PSMD1 mRNA by siRNA was confirmed even in normal cells. The selective cytotoxic effects of PSMD1 depletion on cancer cells were not limited to HCC cells. Massive apoptosis and cell growth inhibition were observed in A549 (p53WT), HT29 (p53MT), and H1299 (p53Null), as evidenced by the CCK-8 assay, annexin V/PI staining, soft agar assay, and spheroid formation assay (Supplementary Fig. S4a–d).

### PSMD1 depletion increases overall protein ubiquitination

**Figure 5:**
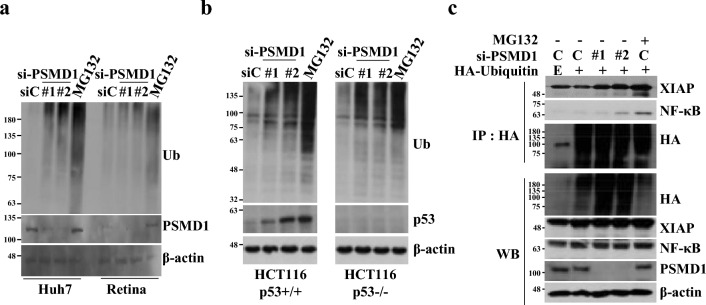
PSMD1 depletion increases overall protein ubiquitination specifically in cancer cells. (**a**,**b**) The impact of PSMD1 depletion on ubiquitination was compared between cancer and normal cells (**a**), or between p53 wt and null cells (**b**) using western blotting. Huh7 and retinal cells or HCT116 p53 + / + and HCT116 p53 − / − cells were transfected with PSMD1 (#1 and #2) and control-siRNA (siC). Three days after siRNA transfection, the levels of ubiquitinated proteins were determined using the ubiquitin antibody. (**c**) 293T cells were transfected with plasmids encoding PSMD1-siRNA or HA-tagged ubiquitin and immunoprecipitated using an antibody against HA, and the immunoprecipitates were probed with antibodies against HA, XIAP, and NFκB. IP and WB indicate immunoprecipitation and western blotting, respectively. Uncropped western blot images corresponding to Fig. 5 can be found in Supplementary Fig. S10.

PSMD1 is a regulatory subunit of the 26S proteasome complex^15–17^. Based on the function of PSMD1, we speculated that its depletion would inhibit the proteasomal activity of the complex, thereby affecting protein ubiquitination and degradation. To investigate the correlation between proteasomal activity, cell survival, and p53 status, we analyzed the total protein ubiquitination pattern in cell lines with definitively comparable parameters for cell survival (Huh7 and retinal cells) and p53 status (HCT116 and its genetically modified counterpart, HCT116 p53-/-). To assess the ubiquitination pattern of total proteins upon PSMD1 depletion, western blotting was performed using an antibody to detect ubiquitinated proteins. We observed that ubiquitination was significantly increased by depletion of PSMD1 (Fig. 5a, b) in cancer cells. Unlike Huh7 cells, ubiquitination was not increased in normal retinal cells (Fig. 5a) or BJ cells (Supplementary. Fig. S5). Additionally, PSMD1 depletion significantly increased protein ubiquitination in both HCT116 cells and HCT116 p53-/- cells, and the extent of increase was not comparable (Fig. 5b). This result indicates that the inhibition of cancer cell survival by PSMD1 depletion was accompanied by an increase in overall protein ubiquitination. Ubiquitination following PSMD1 depletion is verified at the protein level. X-linked inhibitor of apoptosis protein (XIAP) and nuclear factor kappa B (NF-κB) were chosen as representative substrates because the dysregulation of anti-apoptotic factors can potentially enhance apoptosis following PSMD1 depletion. We performed an immunoprecipitation assay using an anti-hemagglutinin (HA) antibody and protein lysates from cells in which HA-ubiquitin was overexpressed and PSMD1 was depleted. Purified immunoprecipitates were probed with antibodies against XIAP and NF-κB. These proteins were detected in the immunoprecipitates at levels similar to those observed in cells treated with the proteasome inhibitor MG132^25^ (Fig. 5c). Considering that the ubiquitination of selected substrates was increased, it can be expected that the inhibition of PSMD1 expression causes defects in the normal ubiquitination-proteasome degradation pathway, resulting in the ubiquitination of numerous proteins.

## Discussion

In this study, we explored the role of PSMD1 in cancer cell ubiquitination and survival. PSMD1 is overexpressed in various human cancer cell lines expressing both wild-type and mutant p53 compared with normal fibroblasts and epithelial cell lines. Using cancer cell lines overexpressing PSMD1, we found that PSMD1 depletion inhibited the removal of ubiquitinated proteins and cancer cell survival.

A previous study reported that p53 degradation is associated with the ability of PSMD1 to regulate breast cancer cell growth^18^. The authors showed that PSMD1 depletion inhibits p53 protein degradation in MCF7 breast cancer cells expressing wild-type p53. Similarly, we observed that p53 was involved in controlling tumor cell growth in our system, as we observed an upregulation of p53 protein levels in Huh7 cells undergoing apoptosis through PSMD1 depletion. We confirmed that the PSMD1-depletion effect that inhibited cancer cell survival also occurred in HCT116 p53 +/+ and HCT116 p53−/− cells, implying that the ability of PSMD1 to regulate cancer cell survival is independent of the p53 status. In accordance with the survival results, ubiquitinated protein accumulation was detected in wild-type and p53-deficient HCT116 cells following PSMD1 depletion. PSMD1 is a component of the 19S regulatory particle^15–17^, which removes ubiquitin from a substrate protein^7^ and delivers the deubiquitinated protein to the 20S core particle for degradation^8^. Therefore, our observation that the inhibition of PSMD1 expression led to increased ubiquitinated proteins implies that ubiquitinated protein degradation is defective under these conditions. We conclude that an increase in ubiquitinated proteins contributes to cancer cell apoptosis.

Ubiquitin-conjugated and ubiquitin-like proteins contribute to the induction of the DNA double-strand break response^26^. Herein, we found that PSMD1 depletion increased the G2M phase, the cell phase accumulated by the DNA damage checkpoint activation, and increased the nuclear level of γ-H2AX, which is a marker for DNA double-strand breaks. We did not elucidate the mechanism underlying cell cycle arrest following PSMD1 depletion. However, considering that proteasome inhibitors, such as MG132, epoxomicin, carfilzomib, and bortezomib, induce ubiquitin-conjugated protein accumulation and eventually lead to DNA damage^14,27^, it is possible that PSMD1 depletion through a dysregulated ubiquitination process causes stress inducing endogenous DNA damage, which can activate the DNA damage checkpoint, induce cell cycle arrest, and ultimately lead to cell death.

No toxicity due to expression inhibition, specifically in normal cells, was found, increasing the value of this protein as a new cancer target. Given its overexpression in cancer and its distinct poor prognostic value, PSMD1 is predicted to have a specific function in cancer cells, unlike normal cells, apart from the common cell survival mechanism in normal and cancer cells. PSMD1 may play a crucial role in maintaining cellular homeostasis by supporting essential ubiquitination processes in cancers with high metabolic activity, thereby contributing to cancer cell survival. Most current proteasome inhibitors focus primarily on targeting the 20S core particle components^28^. Therefore, with relatively few specifically targeting the 19S regulatory particles^28,29^, using PSMD1-targeting inhibitors represents a potential novel therapeutic approach for anticancer treatment. Although the absence of animal experiments limits the direct clinical significance of targeted anticancer treatments, our study provides valuable information regarding the potential role of PSMD1 in cancer. Furthermore, this study emphasizes the need for further investigations to fully elucidate its therapeutic implications.

## Methods

### Cell cultures

HepG2 and Hep3B (hepatocellular carcinoma), HeLa (cervical cancer), HCT116 and HT29 (colorectal cancer), MDA-MB-231 (breast cancer), HT1080 (fibrosarcoma), A549 and H1299 (non-small cell lung cancer), U2OS and SAOS2 (osteosarcoma), Wi38 and IMR90 (normal lung fibroblast), Wi38VA13 (SV40 transformed Wi38), human retinal pigmented epithelial cells, and THLE-2 were purchased from the American Type Culture Collection (ATCC, Manassas, VA, USA). Small airway epithelial cells were purchased from Lonza (CC-2547, Lonza, Switzerland). Huh7 and SNU475 cells (hepatocellular carcinoma) were purchased from the Korean Cell Line Bank (Seoul, South Korea). The cells were cultured as follows: HCT116, MDA-MB-231, Huh7, HT29, H1299, and SNU475 cells in RPMI1640 (LM011-01, Welgene, Daegu, South Korea); HepG2, Hep3B, HeLa, IMR90, Wi38, and Wi38VA13 cells in MEM (LM007-07, Welgene); HT1080 cells in DMEM (LM001-05, Welgene); U2OS and SAOS2 cells in McCoy’s 5A (LM005-01, Welgene); human retinal pigmented epithelial cells in DMEM F12 (11320-033, ATCC); airway small epithelial cells using a small airway epithelial cell growth medium bullet kit (CC-3118, Lonza) and THLE-2 cells in BEGM Bullet Kit (CC3170, Lonza). All cells were grown with 5% CO_2_ in a 37°C incubator. The media were supplemented with 10% (w/v) fetal bovine serum (JR Scientific Inc., Woodland, CA, USA) and 1% (w/v) penicillin/streptomycin (Welgene).

### Patients and tissue samples

HCC and adjacent liver tissue samples were collected from patients diagnosed with HCC who underwent liver surgery between 1992 and 2004 at the Korea Cancer Center Hospital. Frozen tissues from 84 tumor samples, including 33 pairs of adjacent liver tissues, were used. Normal liver tissues were collected from patients who underwent liver resection for tumors with liver metastases from colorectal or rectal cancer. This study was approved by the Institutional Review Board of Korea Cancer Center Hospital, which waived the requirement for informed consent. All procedures involving patient tissues were performed in accordance with relevant institutional guidelines and regulations for human studies under study protocols approved by the Institutional Review Board. The collected clinicopathological information was used to investigate the association between PSMD1 expression and HCC characteristics.

### TCGA data analysis

The relative expression levels of PSMD1 in various human cancers, including HCC, breast carcinoma, and lung and rectal adenocarcinoma, were analyzed using GEPIA (http://gepia2.cancer-pku.cn/#index) based on data from the TCGA and GTEx databases^30^. A box plot was automatically generated using a gene expression analysis category (http://gepia2.cancer-pku.cn/#analysis) from a selected cancer-type dataset. Kaplan–Meier survival curves of patients with liver cancer with various expression levels of PSMD1 were analyzed using the UALCAN platform (https://ualcan.path.uab.edu/se) with data sourced from the TCGA database^31^.

### Real-time PCR

Real-time PCR was used to analyze the relative expression of *PSMD1* in patient tissues and confirm the knockdown efficiency of siRNA in transfected cells. The Tissuescan Liver Cancer cDNA Array was purchased from OriGene (LVRT101, Rockville, MD, USA). The mRNA levels of *PSMD1* and β2-microglobulin (*β2M*)^32^ were measured using real-time PCR using a KAPA SYBR FAST Universal qPCR kit (KK4301, Kapa Biosystems, Inc., Boston, MA, USA) and a CFX96 Detection system (Bio-Rad Laboratories, Inc., Hercules, CA, USA). Of the provided eight normal liver and 24 hepatocellular carcinoma tissues, all normal and 23 cancer tissues, in which the reference gene, *β2M*, was appropriately amplified, were used for analysis. The following primer sequences were used: PSMD1, 5’-ATCCTGCCCCTCTGGAAGTA-3’ (forward), 5’-TGCCTCCAATAGAGAGTGGT-3’ (reverse); and β2‑microglobulin (β2M) forward, 5'‑GTG CTC GCG CTA CTC TCT CT‑3' and reverse, 5'‑CGG CAG GCA TAC TCA TCT TT‑3. Each sample was analyzed in triplicate, and the average cycle threshold (Ct) values were used for subsequent calculations. The relative expression of each tissue against the average expression of normal tissue was analyzed using the comparative threshold cycle (2^−ΔΔCt^) method. Data are presented as the mean ± standard deviation for each group.

### Immunofluorescence staining and live/dead assay

Following transfection with siRNAs, DNA damage was monitored with a γ-H2AX antibody and nuclear counterstaining. Briefly, cells grown on coverslips were fixed in 4% (v/v) paraformaldehyde for 10 min and permeabilized with 0.2% (v/v) Triton X-100 for 15 min at room temperature. The coverslips were blocked with 2.5% (w/v) skim milk in TBS-T (140 mM NaCl, 25 mM Tris pH 7.4, 2.7 mM KCl, and 0.05% (v/v) Tween 20) for 1 h at room temperature, incubated with a primary antibody against γ-H2AX (2577S, Cell Signaling Technology, Denver, MA, USA) for 2 h, and then incubated with an Alexa Fluor 555-conjugated goat anti-rabbit secondary antibody (A21428, Invitrogen, Chicago, IL, USA) for 1 h. The nuclei were counterstained with Hoechst 33258 dye (23491-45-4, Sigma-Aldrich) for 5 min, and the cells were washed with phosphate-buffered saline (PBS) and mounted in mounting solution (90% (w/v) glycerol/ 100 mM Tris, pH 8.8). Fluorescence images were captured using an Axio Imager M2 microscope (Carl Zeiss, Oberkochen, Germany). For quantification, independently transfected cells were analyzed in triplicates. The fluorescence intensity of at least 50 individual cells from each triplicate was calculated using the Image J software.

For live/Dead cell viability assay (Invitrogen, L3224), cells grown on coverslips and transfected with siRNAs for three days were labeled with 2 µM of calcein AM and 4 µM of ethidium homodimer in PBS solution for 30 min at 37°C. After washing with PBS, the cells were mounted, and fluorescence images were captured using a Zeiss Axio Imager M2 microscope.

### Transfection of siRNAs

Human PSMD1-siRNAs were designed using the online software BLOCK-iT™ RNAi Designer (Invitrogen) and synthesized by Genolution (Seoul, South Korea). Cells were grown on 60- or 100-mm culture dishes and transfected with siRNAs using the Lipofectamine RNAiMax reagents (13778-150, Invitrogen), according to the manufacturer’s protocol. The siRNA sequences used were: si-PSMD1#1 5ʹ-CCGCUGGAAUUAUUUCUCUUCUGGAUU-3ʹ, si-PSMD1#2 5ʹ- CAGCCAGUUUGGGUGUAAUUCAUAA-3ʹ, and control-siRNA, 5ʹ-CCUCGUGCCGUUCCAUCAGGUAGUU-3ʹ.

### Clonal survival analysis

Following transfection with control- and PSMD1-siRNAs, cells were seeded in culture dishes at a low density (< 2 × 10^3^ cells per 60-mm dish), grown for seven to 10 days, fixed in 4% (v/v) paraformaldehyde for 10 min, and stained with 0.1% (w/v) crystal violet. The stained colonies were photographed, and survival rates were determined by calculating the ratio of surviving PSMD1-siRNA- versus control-siRNA-transfected colonies.

### Western blotting and ubiquitination analysis

Cells were lysed with RIPA buffer (40 mM Tris–HCl, pH 7.4, 120 mM NaCl, 0.5% (v/v) Nonidet P-40, 0.1% (w/v) sodium dodecyl sulfate, 0.5% (w/v) sodium deoxycholate, and a protease inhibitor cocktail) at 4 °C for 30 min. The cell lysates were centrifuged at 12,000 *rpm* for 20 min at 4 °C. Proteins in the supernatant were resolved using sodium dodecyl sulfate–polyacrylamide gel electrophoresis and transferred to nitrocellulose membranes. The membranes were blocked with 5% (w/v) skim milk in TBS-T buffer and incubated with the primary antibody for 1 h. After three washes with TBS-T buffer, the membranes were incubated with a horseradish peroxidase-conjugated secondary antibody (A120-101P or A90-116P, Bethyl Laboratories, Montgomery, TX, USA) at room temperature for 1 h. Proteins were detected using a western blotting luminol reagent (sc-2048, Santa Cruz Biotechnology).

To analyze the ubiquitination of proteins, 293 T cells were transfected with plasmids encoding HA-tagged ubiquitin (a gift from Edward Yeh, Addgene plasmid # 18712), further transfected with PSMD1-siRNA, treated with 10 μM MG132, and harvested. The cells were lysed with RIPA buffer. The cell lysates were incubated with HA antibody overnight at 4°C, treated with Protein G Sepharose 4 Fast Flow (17-0618-01, Miltenyi Biotech, Bergisch Gladbach, Germany), and incubated at 4 °C for 4 h. Beads were collected through centrifugation, washed three times with RIPA buffer, and boiled in sodium dodecyl sulfate–polyacrylamide gel electrophoresis sample buffer for 5 min. The resulting supernatants were subjected to western blot analysis. Antibodies used for western blotting were as follows: PSMD1 (sc-166038, Santa Cruz Biotechnology), β-actin (sc-47778, Santa Cruz Biotechnology), NF-κB (#3034, Cell Signaling Technology), γ-H2AX, PARP (9542S, Cell Signaling Technology), p53 (SC-126, Santa Cruz Biotechnology), XIAP (610762, BD Biosciences, San Jose, CA, USA), HA antibody (SC-805, Santa Cruz Biotechnology), and ubiquitin (sc-8017, Santa Cruz Biotechnology).

### Apoptosis and cell cycle analyses

Apoptosis was determined using an APC-Annexin V (550475, BD Biosciences) according to the manufacturer’s instructions. Two days after transfection with PSMD1 or control-siRNA, cells were harvested and stained with PI (P4170, Sigma-Aldrich) and APC-annexin V for 15 min at room temperature. Annexin/PI-positive cells were analyzed using flow cytometry (Accuri C6 Plus, BD Biosciences) within 1 h. To monitor the cell cycle profile following siRNA transfection, cells were fixed with 100% (v/v) ethanol for 60 min at 4°C and then stained with 40 μg/mL PI at room temperature for 30 min. The DNA content of the stained cells was analyzed and plotted on a histogram using FACS (FACSCalibur; BD Biosciences).

### Cell proliferation assay

Cell proliferation was determined using a D-Plus™ CCK kit (CCK-3000, Donginbio, Seoul, Korea). Cells transfected with PSMD1-siRNA were seeded in 96-well plates at 1 × 10^3^ cells/well. Two days after seeding, the cells were treated with the D-Plus™ CCK reagent and incubated for 2 h at 37 °C. The absorbance was measured at 450 nm using a Spectra Max190 (Molecular Devices, CA, USA) and used to calculate the proportion of viable cells.

### Spheroid formation

Culture plates were coated with a 10% (w/v) poly-2-hydroxyethyl methacrylate (poly-HEMA; sc-253284, Santa Cruz Biotechnology) solution, which was dissolved in ethanol and dried. Cells were seeded on the coated plates and incubated with 5% (v/v) CO_2_ at 37 °C. The resulting spheroids were photographed under a light microscope (× 40).

### Growth on soft agar

Bottom- (0.5% (w/v)) and top-agarose (0.35% (w/v)) solutions were prepared in RPMI-1640 medium containing 10% (w/v) fetal bovine serum. Cancer cells (1500 cells/well) were mixed with the top-agarose solution, layered onto a 24-well plate prepared with bottom-agarose, and incubated with 5% (v/v) CO_2_ at 37 °C until colonies became visible. The colonies on the agar plates were stained with 0.05% (w/v) crystal violet and counted to evaluate anchorage-independent cancer cell growth.

### Statistical analysis

Statistical analyses of survival and apoptosis were performed using SPSS software (v.23.0, IBM Corp., Armonk, NY, USA). Statistical differences between the experimental and control groups were analyzed using one-way analysis of variance with Tukey’s post hoc HSD test. Statistical significance was reached when the *p*-value was less than 0.05. All bar graphs are presented as mean ± standard deviation.

### Supplementary Information

Supplementary Figures.

## Data Availability

All data are available under reasonable request by contacting the corresponding author.
