# Author Correction: Depletion of proteasome subunit PSMD1 induces cancer cell death via protein ubiquitination and DNA damage, irrespective of p53 status

**DOI:** 10.1038/s41598-024-60634-1

**Published:** 2024-05-03

**Authors:** Mi-Yeun Kim, Eun-Ran Park, Eung-Ho Cho, Sun-Hoo Park, Chul Ju Han, Sang-Bum Kim, Man Bock Gu, Hyun-Jin Shin, Kee-Ho Lee

**Affiliations:** 1 Division of Radiation Biomedical Research, Korea Institute of Radiological and Medical Sciences, Korea University, 75, Nowon-Ro, Nowon-Gu, Seoul, 01812 South Korea; 2 Department of Biotechnology, College of Life Sciences and Biotechnology, Korea University, Seoul, South Korea; 3 Department of Surgery, Division of Radiological and Clinical Research, Korea Institute of Radiological and Medical Sciences, Seoul, South Korea; 4 Department of Pathology, Division of Radiological and Clinical Research, Korea Institute of Radiological and Medical Sciences, Seoul, South Korea; 5 Department of Internal Medicine, Division of Radiological and Clinical Research, Korea Institute of Radiological and Medical Sciences, Seoul, South Korea

Correction to: *Scientific Reports* 10.1038/s41598-024-58215-3, published online 05 April 2024

The original version of this Article contained errors in the Methods section.

In the Methods section, under the subheading ‘Patients and tissue samples’,

“This study was approved by the Institutional Review Board of Korea Cancer Center Hospital, which waived the requirement for informed consent (No. 012-11-001).”

now reads:

“This study was approved by the Institutional Review Board of Korea Cancer Center Hospital, which waived the requirement for informed consent.”

“All procedures involving patient participants were performed in accordance with relevant institutional guidelines and regulations for human studies under study protocols approved by the Institutional Review Board.”

now reads:

“All procedures involving patient tissues were performed in accordance with relevant institutional guidelines and regulations for human studies under study protocols approved by the Institutional Review Board.”

The original Article has been corrected.

