# Supplementary material for: Depletion of proteasome subunit PSMD1 induces cancer cell death via protein ubiquitination and DNA damage, irrespective of p53 status
Source: Sci Rep. 2024 Apr 5;14:7997. doi: 10.1038/s41598-024-58215-3 (PMC10997673; doi:10.1038/s41598-024-58215-3)

Supplementary Information

Depletion of proteasome subunit PSMD1 induces cancer cell death via protein ubiquitination and DNA damage, irrespective of p53 status

Mi-Yeun Kim[1,2]*, Eun-Ran Park[1], Eung-Ho Cho[3], Sun-Hoo Park[4], Chul Ju Han[5] , Sang-Bum Kim[3], Man Bock Gu[2], Hyun-Jin Shin[1]¶, and Kee-Ho Lee[1]¶

[1]Division of Radiation Biomedical Research, Korea Institute of Radiological and Medical Sciences, [2]Department of Biotechnology, College of Life Sciences and Biotechnology, Korea University, Seoul, Korea, Department of Surgery[3], Pathology[4], and Internal Medicine[5], Division of Radiological and Clinical Research, Korea Institute of Radiological and Medical Sciences, Seoul, Korea

¶Correspondence: Kee-Ho Lee, Division of Radiation Biomedical Research, Korea Institute of Radiological and Medical Sciences, 75, Nowon-Ro, Nowon-Gu, Seoul, 01812, Korea, Phone: 82-2-970-1312, E-mail: khleeby@naver.com; Hyun-Jin Shin, Division of Radiation Biomedical Research, Korea Institute of Radiological and Medical Sciences, 75, Nowon-Ro, Nowon-Gu, Seoul, 01812, Korea, Phone: 82-2-970-1658, E-mail: hjshin@kirams.re.kr

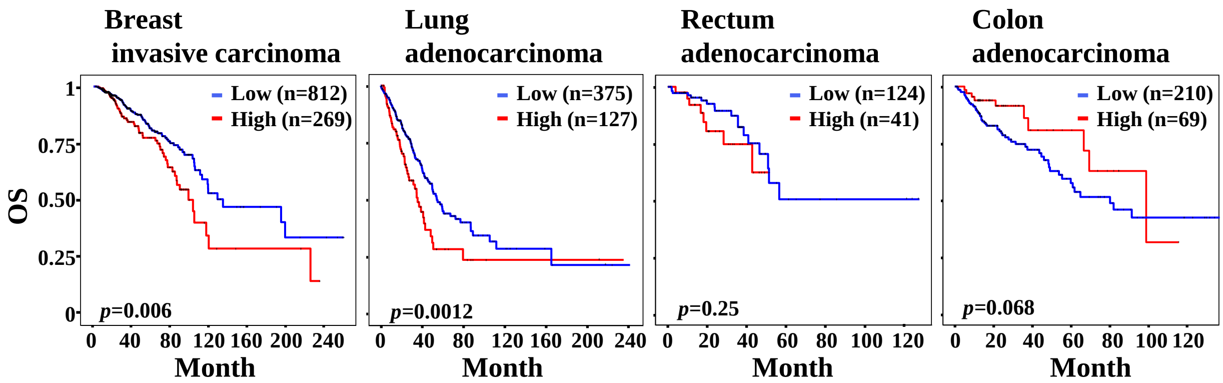

Supplementary Fig. S1 Kaplan–Meier survival curves of the indicated cancers were analyzed based on the level of PSMD1 expression using the UALCAN platform.

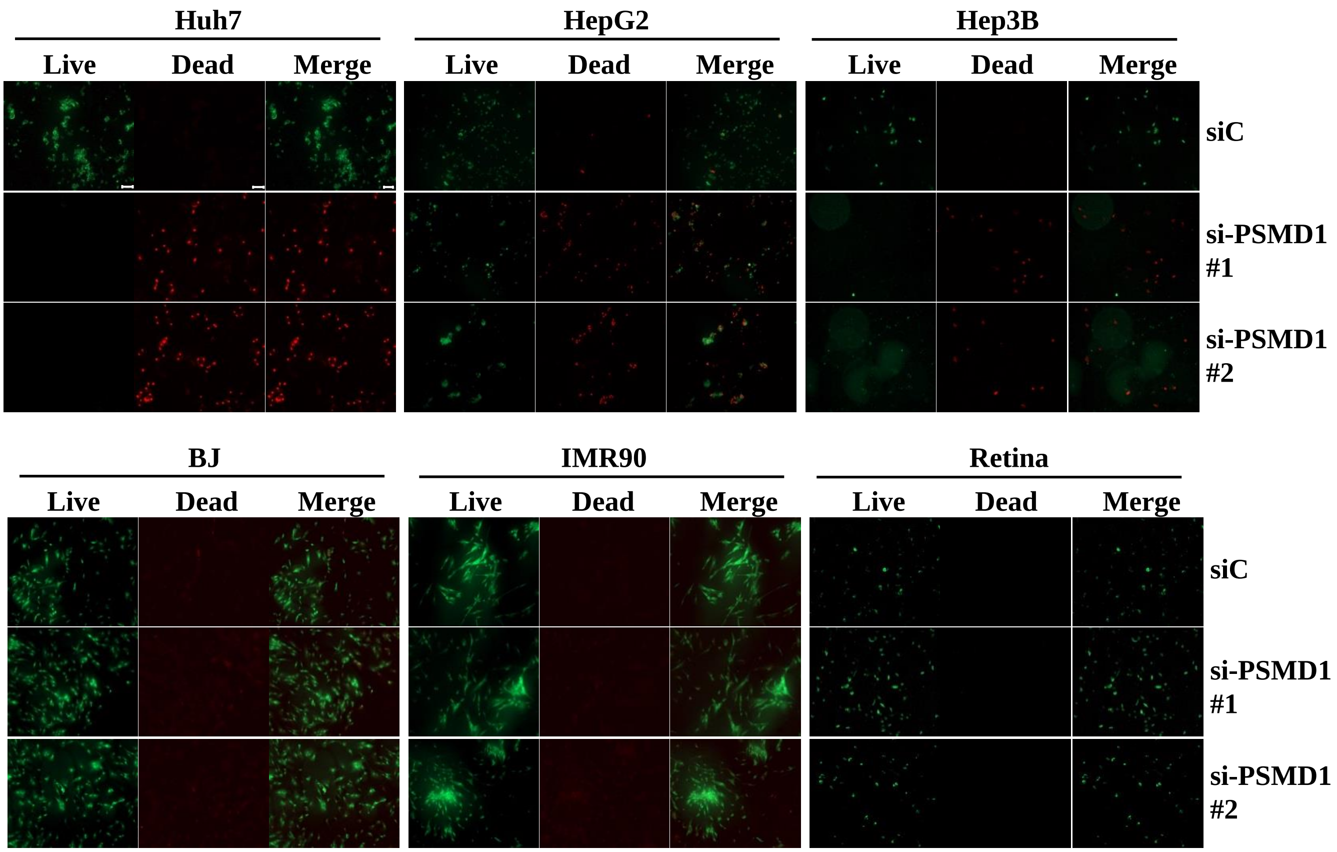

Supplementary Fig. S2. Representative confocal images of live/dead cell assays. This image shows an individual picture of the merged data in Fig. 4b.

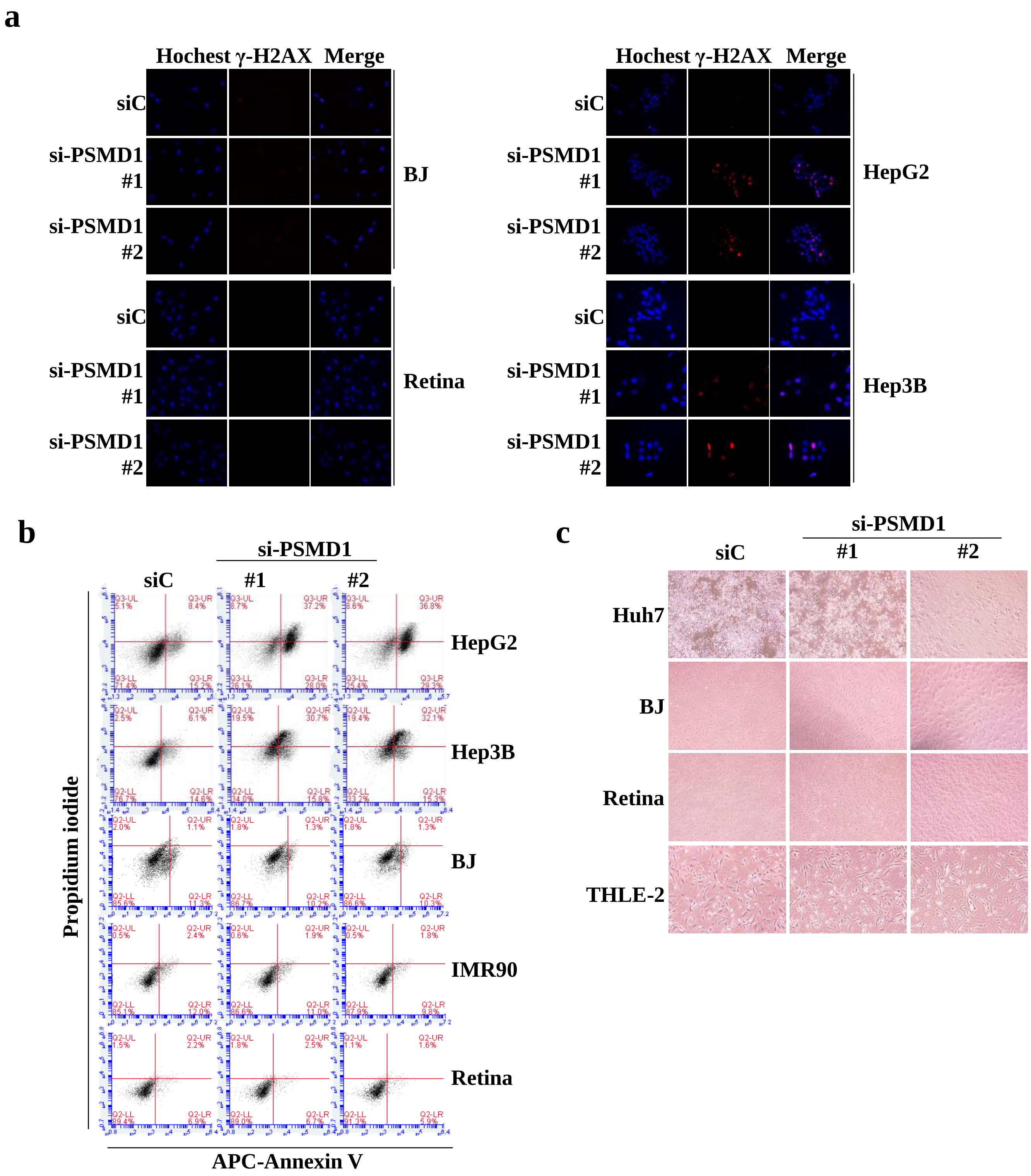

Supplementary Fig. S3. (a) The increased DNA damage by PSMD1 depletion in cancer cells and normal cells was analyzed by staining with the γ-H2AX antibody. Cells were prepared three days after transfection. Hoechst dye was used to counterstain the nuclei, and the stained cells were visualized under a fluorescence microscope (×40). (b) Representative scatter plot with the quadrants used for the bar graph in the lower panel of Fig. 4c. (c) Representative light microscopy image (×40) of PSMD1-transfected cells.

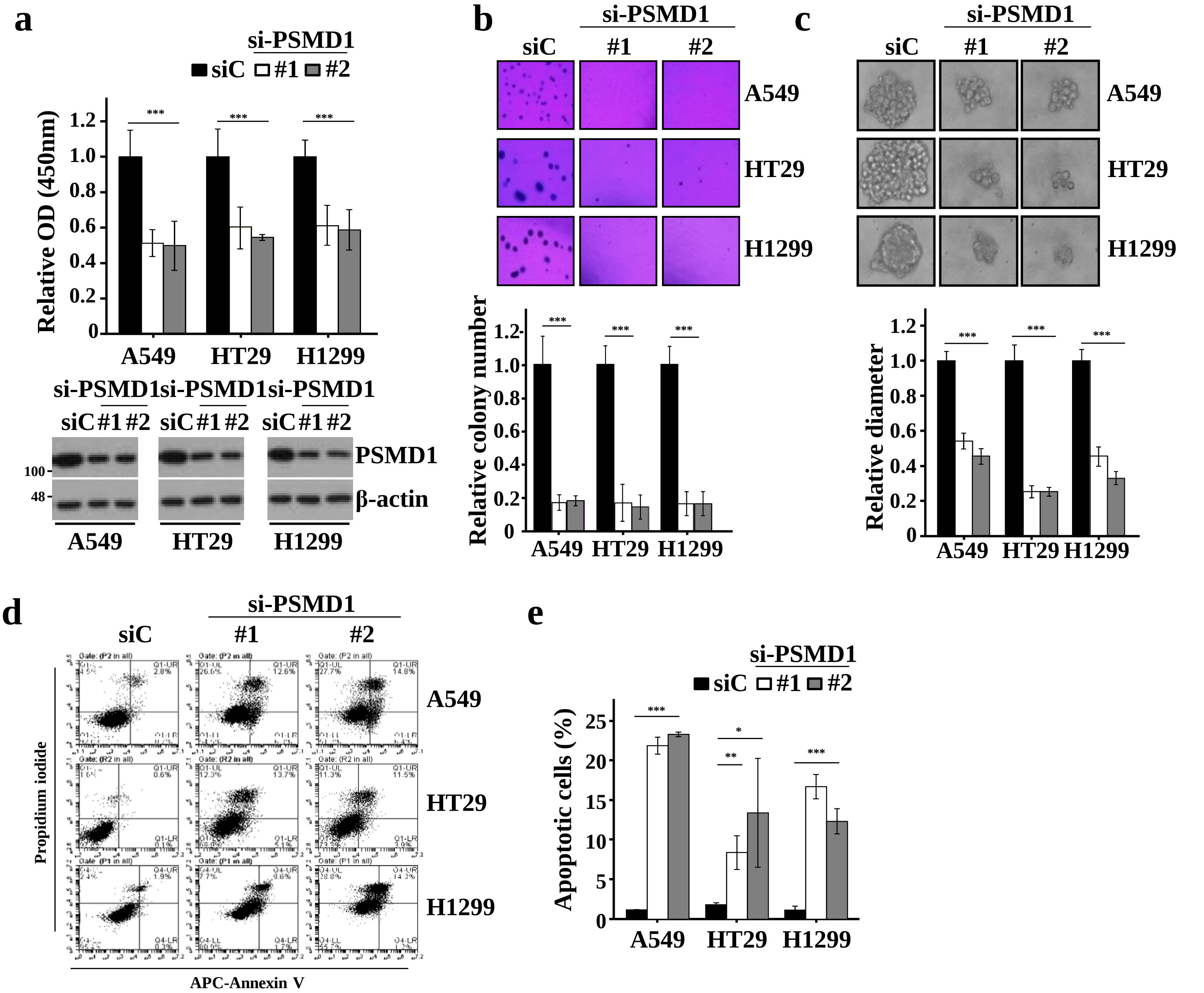

Supplementary Fig. S4 (a–e) A549, HT29, and H1299 cells were transfected with PSMD1- (#1 and #2)- or control-siRNA (SiC). The effects of PSMD1 depletion on cell proliferation (determined using CCK-8 assay) (a), colony formation on soft agar (b), spheroid formation on poly HEMA-coated plates (c), and apoptosis (determined using Annexin V/PI staining) (d) were examined. Images of cells were captured under a light microscope, and the relative proliferation rate (a), colony number (b), and sphere diameter (d) compared with those of control-siRNA-transfected cells, and the percentage of cell population from total cells (c) were plotted as bar graphs from triplicate data.

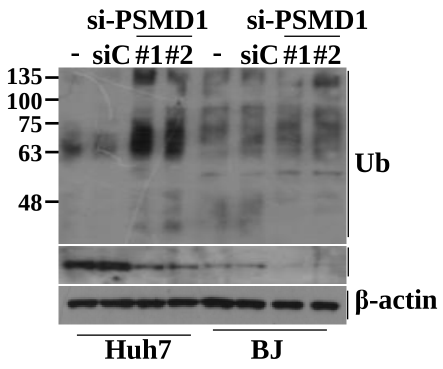

Supplementary Fig. S5. Effect of PSMD1 depletion on ubiquitination in Huh7 and BJ cells was compared. Protein ubiquitination was determined three days after siRNA transfection.

Figure 1d / PSMD1

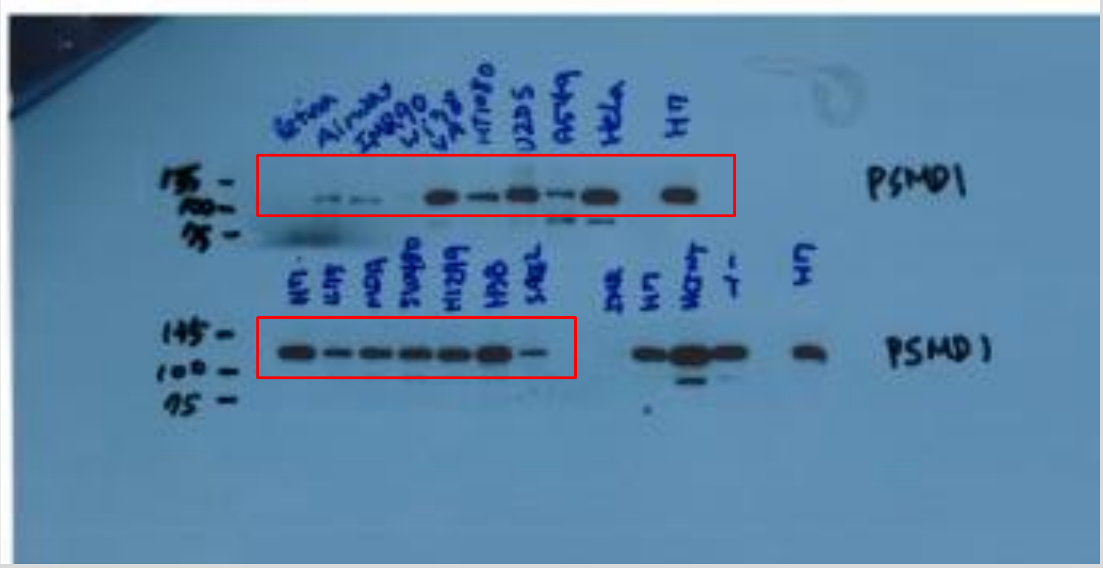

Figure 1d / β-actin

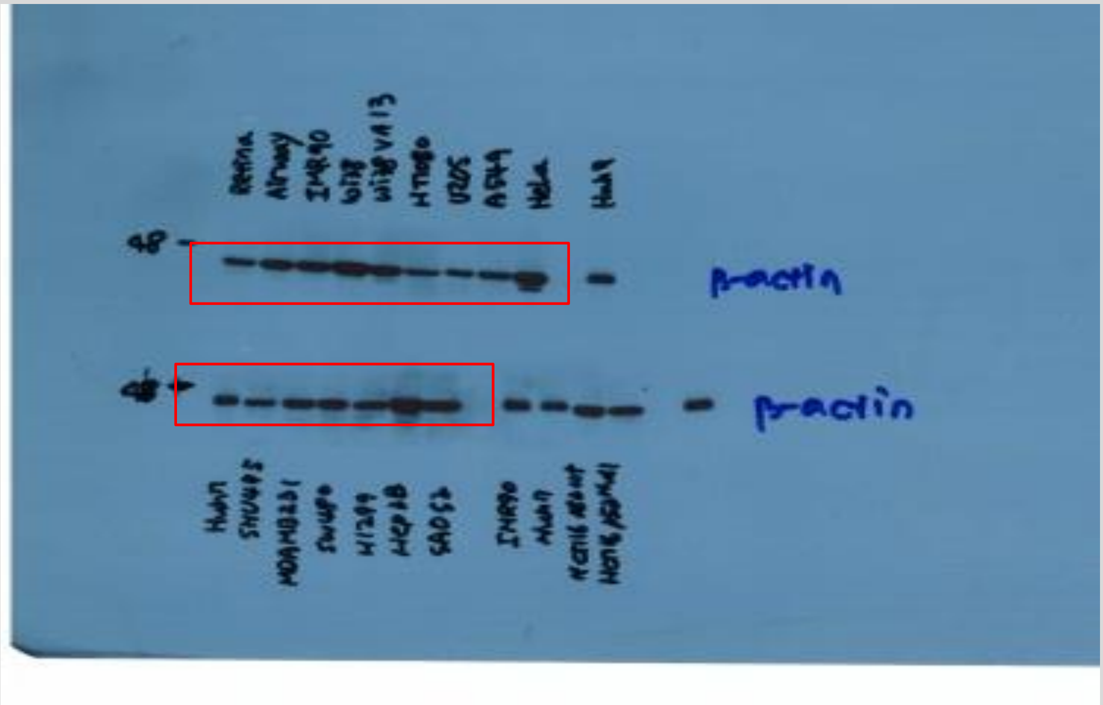

Supplementary Fig. S6. The uncropped original image in Fig. 1.

Figure 2a / PSDM1, β-actin

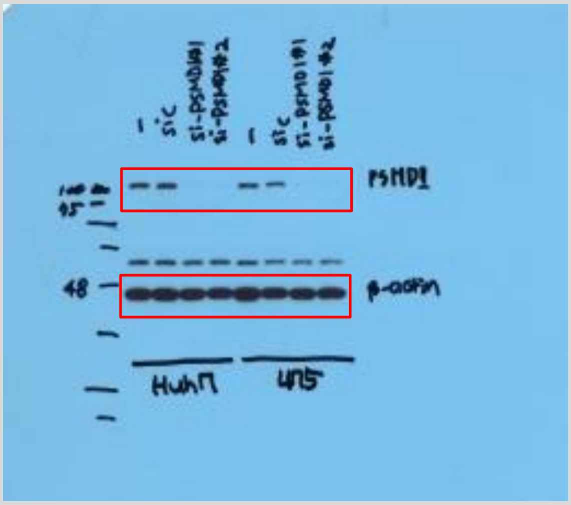

Figure 2c / PARP

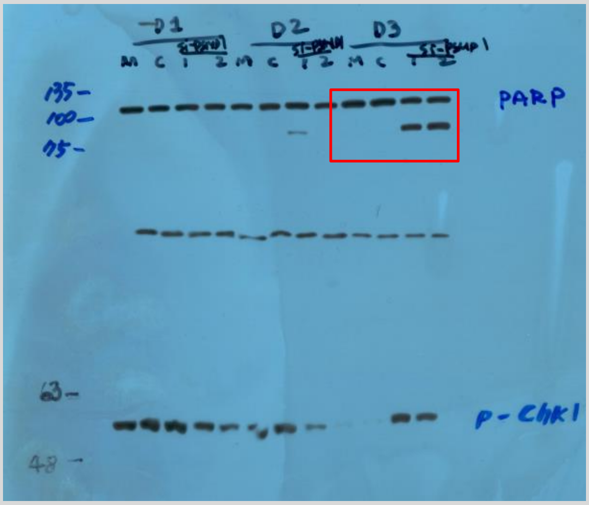

Figure 2c / PSMD1, p53

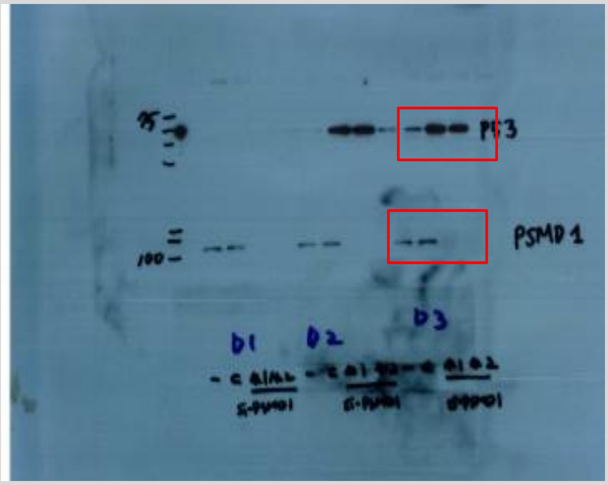

Figure 2c / c-caspase3

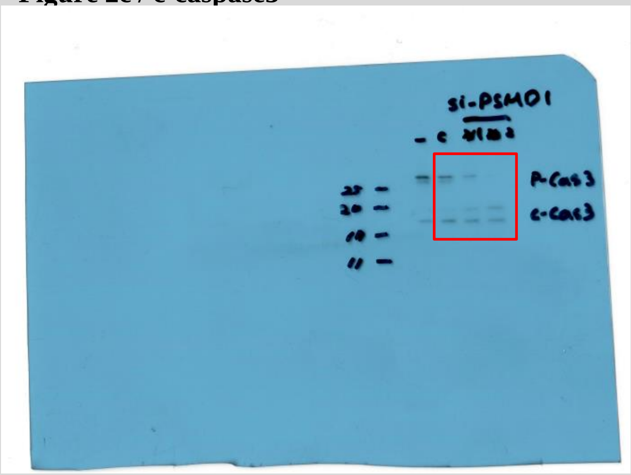

Figure 2c / β-actin, γ-H2AX

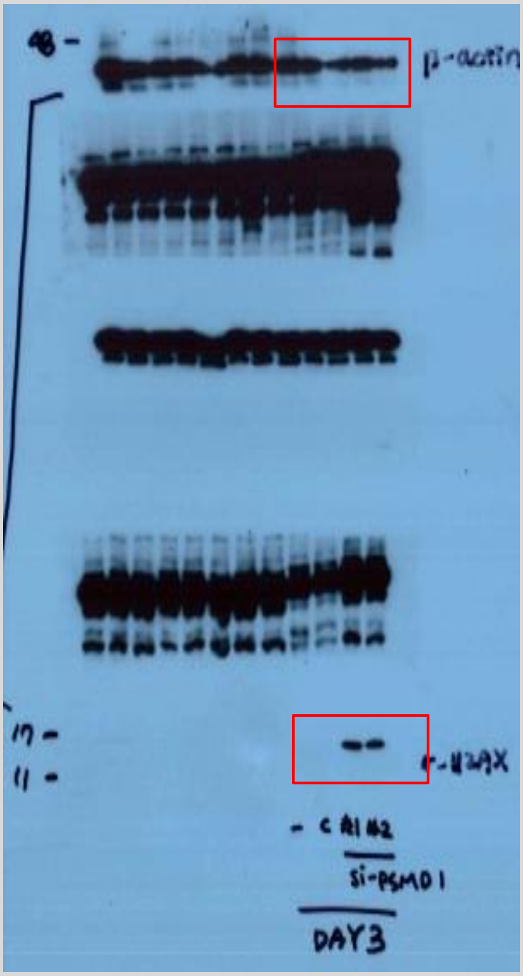

Supplementary Fig. S7. The uncropped original image in Fig. 2.

Figure 3a / H1299, HT29, HeLa /PSMD1, β-actin

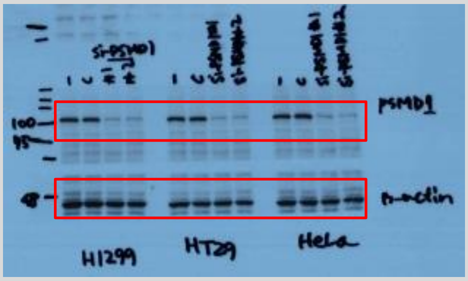

Figure 3a / MDAMB231 , HT1080 /PSMD1, β-actin

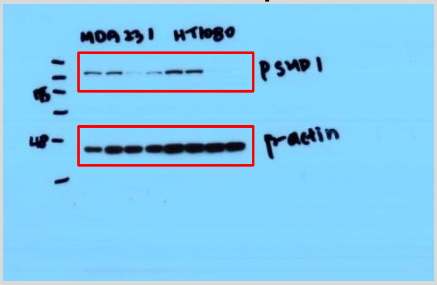

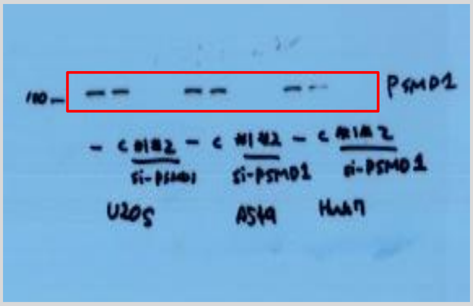

Figure 3a / SAOS2, Hep3B, SW480 /β-actin

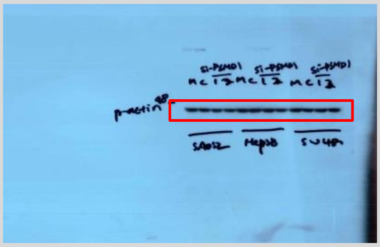

Figure 3a / SAOS2, Hep3B, SW480 / PSMD1

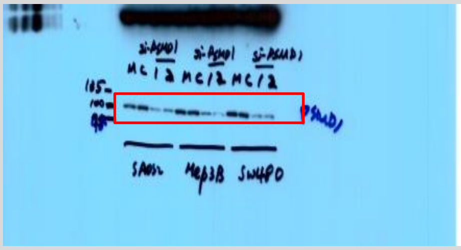

Figure 3a / U2OS, A549 /PSMD1, β-actin

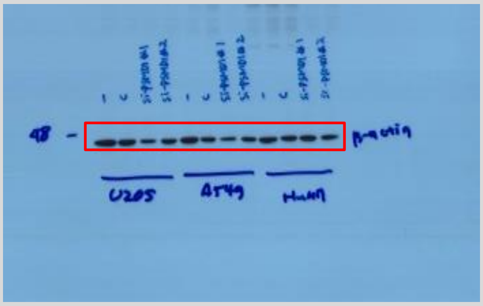

Figure 3a / HepG2 / PSMD1, β-actin

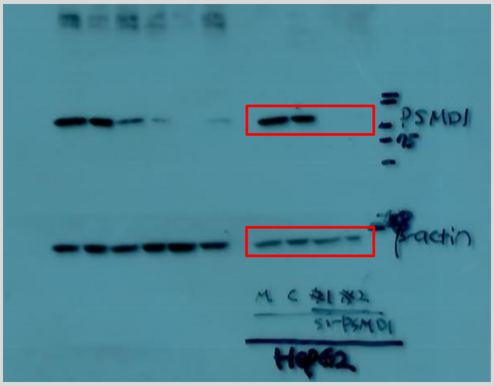

Figure 3c/ PSMD1, β-actin

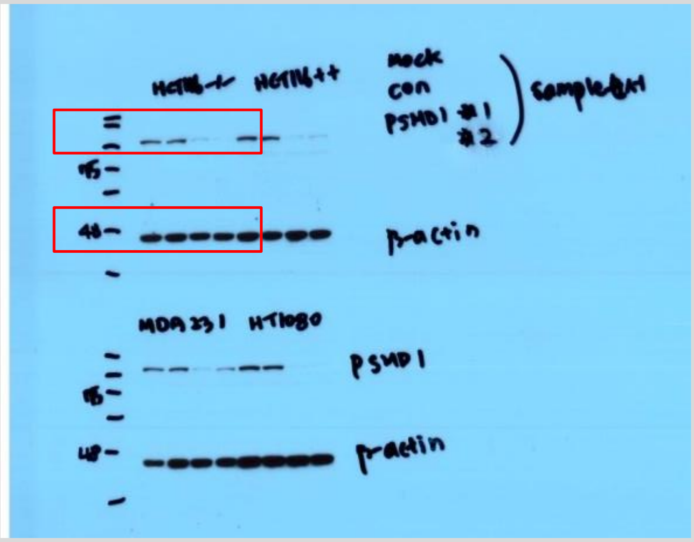

Supplementary Fig. S8. The uncropped original image in Fig. 3.

Figure 4d / PARP, β-actin, PSMD1

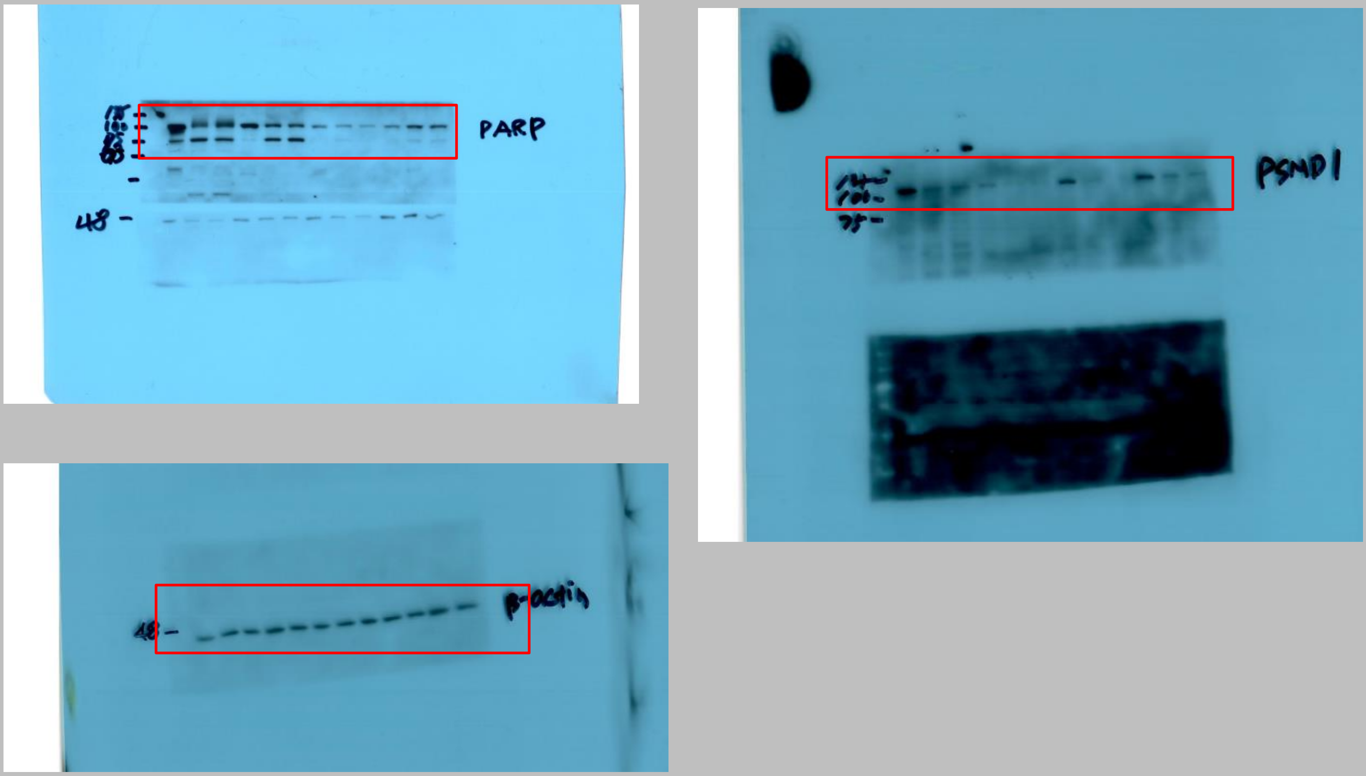

Supplementary Fig. S9. The uncropped original image in Fig. 4.

Figure 5a / Ub

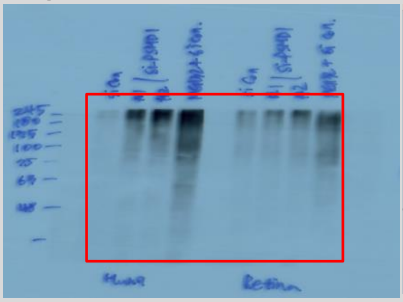

Figure 5a / PSMD1

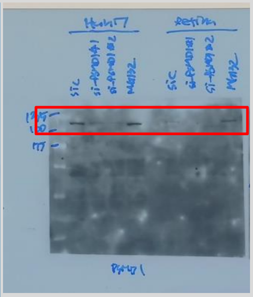

Figure 5a / β-actin

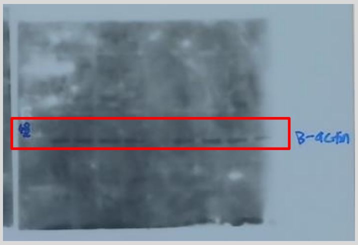

Figure 5b / Ub

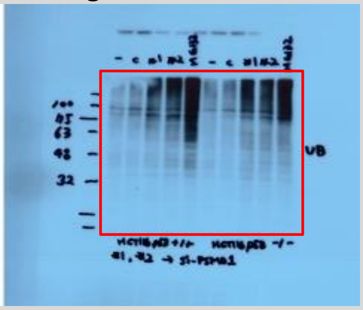

Figure 5b / β-actin

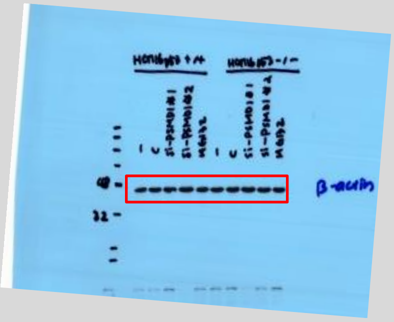

Figure 5b / p53

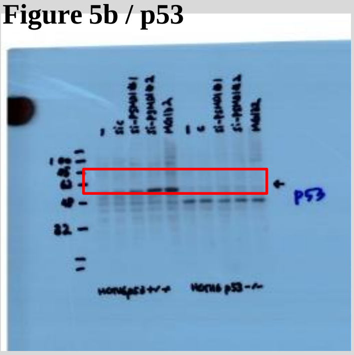

Figure 5c / NF-κB (IP, WB)

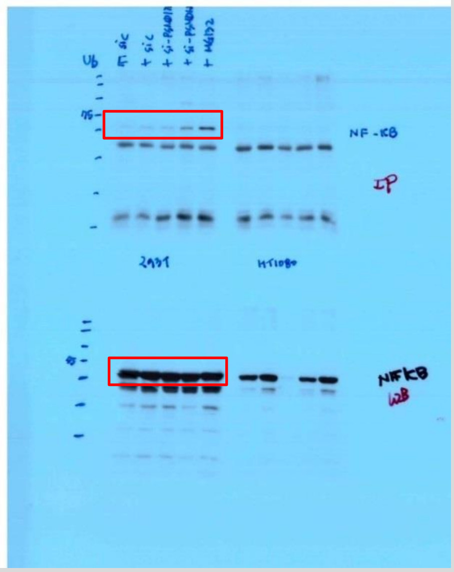

Figure 5c / PSMD1 (IP, WB)

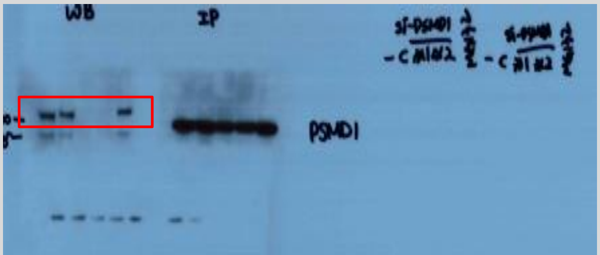

Figure 5c / XIAP (IP, WB) ,β-actin

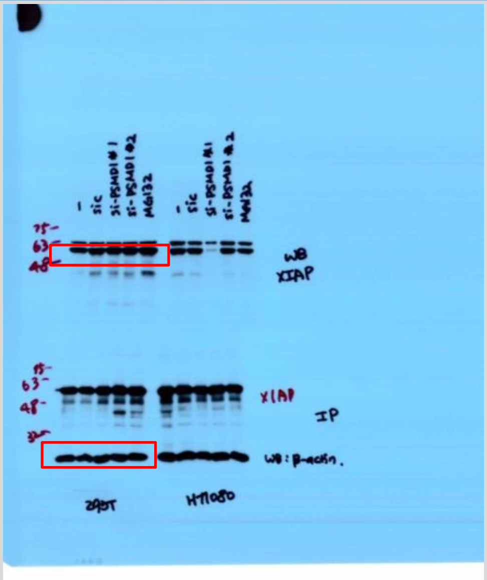

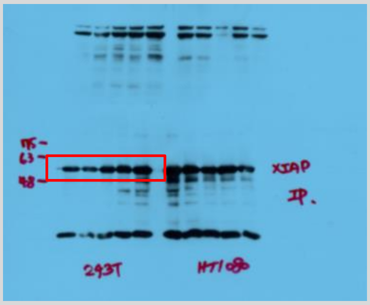

Figure 5c / HA (IP, WB)

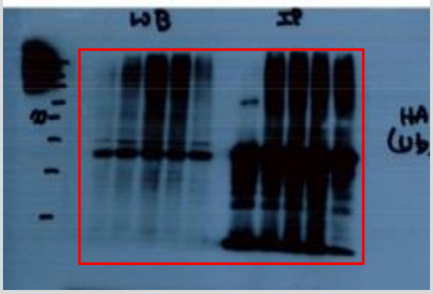

Supplementary Fig. S10. The uncropped original image in Fig. 5.

Figure S4a / PSMD1, β-actin

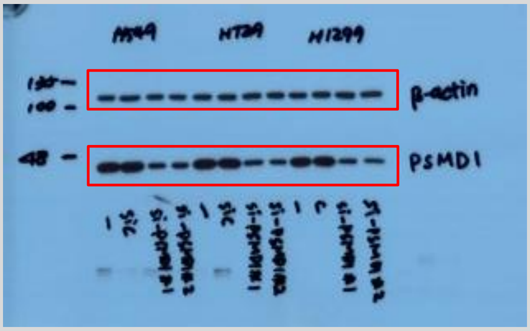

Figure S5 / PSMD1

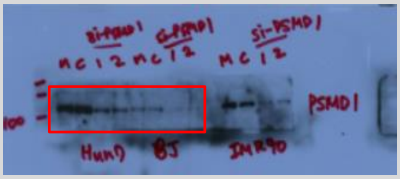

Figure 4d / Ubiquitin

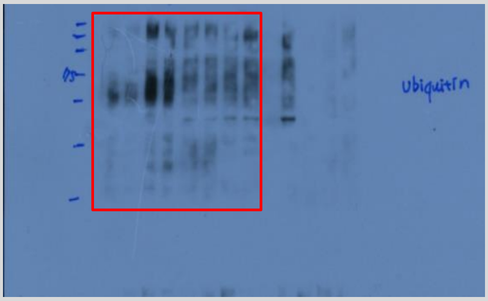

Figure 4d / β-actin

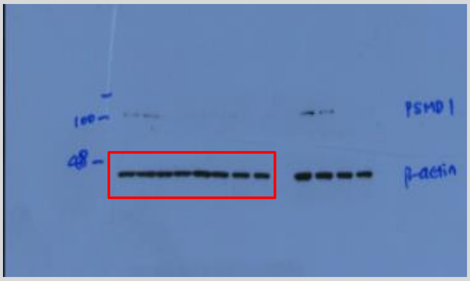

Supplement: Supplementary file 1 — Supplementary Figures. [file 41598_2024_58215_MOESM1_ESM.pdf]
